# Linking Vegetable Per- and Polyfluoroalkyl Substance Accumulation with Root Chemical Traits

**DOI:** 10.1021/acsenvironau.5c00184

**Published:** 2025-12-08

**Authors:** Chun Cao, Qian Huo, Qianhui Tang, Yifan Guo, Liang Zeng, Yao Cheng, Guomao Zheng, Biwei Yang, Junjian Wang

**Affiliations:** † College of Geography and Environmental Science, 12435 Northwest Normal University, Lanzhou, Gansu 730070, China; ‡ Key Laboratory of Resource Environment and Sustainable Development of Oasis, Lanzhou, Gansu 730070, China; § State Key Laboratory of Soil Pollution Control and Safety, 255310 Southern University of Science and Technology, Shenzhen, Guangdong 518055, China; ∥ Guangdong Provincial Key Laboratory of Soil and Groundwater Pollution Control, School of Environmental Science and Engineering, Southern University of Science and Technology, Shenzhen, Guangdong 518055, China; ⊥ State Environmental Protection Key Laboratory of Integrated Surface Water-Groundwater Pollution Control, School of Environmental Science and Engineering, Southern University of Science and Technology, Shenzhen, Guangdong 518055, China; a Engineering Research Center for Ecological and Environmental Damage Assessment of Gansu Province, Lanzhou, Gansu 730070, China

**Keywords:** perfluoroalkyl and polyfluoroalkyl substances, water-extractable organic matter, vegetable accumulation characteristics, soil-to-plant migration, health risk assessment

## Abstract

Per- and polyfluoroalkyl substances (PFASs) are ubiquitous, persistent organic pollutants increasingly detected in food crops, yet their accumulation capacities and regulatory factors across various plant species remain poorly resolved. Here, we investigated the bioaccumulation patterns of PFAS in 20 vegetable species and their relations with root chemical traits in farmland irrigated with treated wastewater. Leafy vegetables (e.g., *Lactuca sativa* and *Spinacia oleracea*) accumulated substantially higher PFAS concentrations (mean: 9.24 ng/g) than the root vegetable *Daucus carota*, with the short-chain perfluorobutanoic acid (PFBA) identified as the dominant species for all vegetables. PFBA showed the strongest mobility and tended to accumulate in edible aerial tissues of leafy vegetables, whereas long-chain PFASs were largely retained in roots. Across vegetable species, root PFBA concentration increased with the proportion of alkyl carbon and decreased with the proportion of *O*-alkyl carbon in roots, whereas the long-chain perfluorononanoic acid concentration increased with dissolved organic carbon concentration in roots. PFAS exposure could be decreased by up to 90% by consuming low-concentration vegetable varieties instead of high-concentration ones. These findings highlight the critical role of plant traits and rhizosphere chemistry in governing PFAS uptake pathways and suggest that crop selection and rhizosphere management can inform risk mitigation.

## Graphical abstract

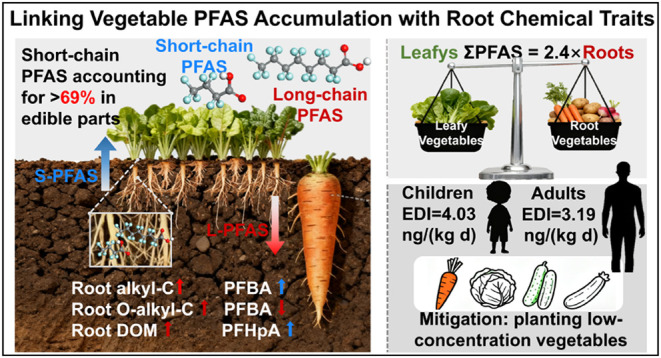

## 1 Introduction

Per- and polyfluoroalkyl substances (PFAS) are synthetic, highly fluorinated organic compounds known for their chemical and thermal stability as well as hydrophobic and oleophobic properties. However, these same characteristics contribute to their environmental persistence and long-range transport via atmospheric deposition, surface runoff, and leaching.^1–3^ Combined with their potential ecological toxicity and human health risks,^1,3–7^ PFAS are now recognized as globally concerning emerging contaminants. In agricultural systems, PFAS introduced through raw or treated wastewater irrigation can accumulate in soils and subsequently enter the food chain via crop uptake. A deeper understanding of PFAS accumulation in edible plants grown in historically contaminated farmland is urgently needed to assess human exposure risks.

PFAS accumulation in plants is influenced by three main factors: PFAS physicochemical properties (e.g., functional group type and carbon chain length), environmental conditions (e.g., soil organic carbon content), and plant-specific traits (e.g., transpiration rate).^3–5^ Among these, carbon chain length is especially important: short-chain PFAS are more water-soluble and mobile, while long-chain PFAS are more likely to accumulate in lipid-rich tissues.^5,8^ Given that roots are the primary interface between soil and plant, the chemical traits of the root systems and the root-derived dissolved organic matter (DOM) they release are, therefore, central to determining PFAS bioavailability and translocation into edible plant parts.

Root chemical traits, including both structural components and water-soluble organic fractions (i.e., DOM), are expected to influence PFAS uptake at the root–soil interface. Because PFAS tend to accumulate in lipid-containing tissues,^5,8^ vegetables with higher lipid content (a key source of alkyl carbon) may exhibit stronger PFAS binding than surrounding soil organic matter, potentially leading to enhanced accumulation. Indeed, He et al.^9^ recently reported that root PFAS bioconcentration factors were strongly correlated with root lipid content across seven weed species, whereas correlations with root morphological traits were weak or insignificant. Moreover, root-derived DOM may exert both facilitative and inhibitory effects on the PFAS behavior at the root–soil interface. On the one hand, DOM can enhance PFAS bioavailability through competitive binding^10–12^ and rhizosphere microbial regulation.^11,13^ On the other hand, DOM can suppress PFAS mobility via hydrophobic complexation,^14^ colloidal coprecipitation,^11^ or macromolecular blockage of membrane transport.^11,15^ These dual roles, evident in contrasting observations such as elevated PFAS uptake in lettuce versus suppressed transfer in wheat,^11,15^ are linked to the molecular weight and functional group heterogeneity of DOM.^11,15,16^ Despite increasing attention, the regulatory role of root chemical traits in regulating PFAS accumulation across crop species remains poorly resolved.

Here, we conducted controlled experiments using soils from farmland exposed to over 50 years of treated wastewater irrigation. Twenty vegetable varieties spanning four categories (leafy, fruit, root, and tuber vegetables) were cultivated to explore PFAS soil–plant transfer mechanisms. Specifically, we aimed to (1) quantify PFAS translocation (TF_ar_) and bioaccumulation factors (BAF_rs_ for roots, BAF_es_ for edible parts) across vegetable species; (2) integrate ^13^C nuclear magnetic resonance spectroscopy (^13^C NMR) and fluorescence excitation–emission matrix (EEMs) to evaluate the regulatory role of root chemical traits in PFAS accumulation; and (3) assess estimated daily intake (EDI) of PFAS for children and adults to support risk-based planting strategies. Our findings reveal interspecific variability in PFAS accumulation and highlight the regulatory role of root chemistry, offering insights for mitigating PFAS risks in agroecosystems.

## 2 Materials and Methods

### 2.1 Study Site

Baiyin City, located in central Gansu Province (Figure S1a), is a historically important nonferrous metal industrial base. Since the 1960s, ∼76% of local farmland has been irrigated with treated industrial wastewater from the Dongdagou and Xidagou Channels. This practice has led to contamination by heavy metals and organic compounds, contributing to soil degradation and designation of the area as a national priority control zone.^7,17–20^ The current study was conducted during the 2018 growing season (April to September) in the Dongdagou River Basin (36.48°N, 104.31°E).

### 2.2 Soil and Vegetable Sampling

Surface soils from a local farmland without artificial PFAS addition were well mixed and used to establish a pot experiment (27.0 ± 0.5 kg soil per pot) in an open-field farmland environment. Twenty vegetable species across four categories (11 leafy, 7 fruit, 1 root, and 1 tuber vegetables) were cultivated (each species with four replicates) using certified seeds (germination >95%). A photograph of the pot experiment setup is provided in Figure S1b. The 11 leafy vegetable included: Chinese cabbage (*Brassica rapa* L. subsp. *pekinensis*), spinach (*Spinacia oleracea* L.), shallot (*Allium ascalonicum* L.), Chinese chives (*Allium tuberosum* Rottler ex Spreng), and cabbage (*Brassica oleracea* var. *capitata*), celery (*Apium graveolens* L.), and Chinese lettuce (*Lactuca sativa* var. *ramose*), garland chrysanthemum (*Chrysanthemum coronarium* L.), coriander (*Coriandrum sativum* L.), and leafy lettuce (*Lactuca sativa* L. var. *longifolia*), and rape (*B. rapa* L. subsp. *chinensis*). The 7 fruit vegetables included cucumber (*Cucumis sativus* L.), kidney bean (*Phaseolus vulgaris* L.), pepper (*Capsicum annuum* L.), winged bean (*Vigna unguiculata* (L.) Walp.), eggplant (*Solanum melongena* L.), tomato (*Solanum lycopersicum* L.), and zucchini (*Cucurbita pepo* L.). The root vegetable was carrot (*Daucus carota* L.), and the tuber vegetable was potato (*Solanum tuberosum* L.). Vegetable species were selected to represent major dietary categories (leafy, fruit, root, and tuber vegetables) based on local consumption patterns and their potential for PFAS accumulation. The predominance of leafy vegetables reflects their high dietary intake and previously reported susceptibility to contaminant uptake, while root and tuber vegetables were included to represent crops with underground edible tissues. During the experimental growing period (April to September), no fertilizers were applied, and irrigation was maintained to keep soil moisture at 70–80% of field capacity using bottled water. Detailed information on the vegetable varieties is provided in Table S1.

Sample collection and processing followed ISO 11464 standards. For soils, original bulk soils and postcultivation rhizosphere soils (<2 mm from roots) were air-dried, ground to pass through a 100-mesh sieve, and stored in brown glass bottles at −20 °C. For plants, samples were divided into roots, aerial parts (stems and leaves), fruits (if available), and tubers (if available). Note that the edible part refers to the aerial part for leafy vegetables, the fruit for fruit vegetables, the root for the root vegetable, and the tuber for the tuber vegetable. Samples were split for two sets of analyses. For PFAS analysis, samples were washed using ultrapure water, then freeze-dried (prefrozen at −80 °C), sieved to 80 mesh, and stored in inert containers. For DOM and ^13^C NMR characterization, samples were rinsed with deionized water, oven-dried at 60 °C to constant weight, homogenized, and sieved for further analysis.

### 2.3 Materials and Reagents

A high-purity standard mixture (≥98%, Wellington Laboratories) containing 30 target PFAS compounds was used, including 11 long-chain PFAS (L-PFAAs), 7 short-chain PFAS (S-PFAAs), and 12 emerging PFAS (E-PFAS). Twenty-two isotopically labeled compounds were employed as surrogate standards and internal standards. Details on all analytes and reagents are listed in Tables S2 and S3 and Text S1.

### 2.4 PFAS Extraction and Quantification

PFAS were extracted from soil and plant samples by using ultrasonic extraction. For soils, 2.0 g of dry soil was spiked with 2 ng of surrogate standard and extracted with 1% NH_4_OH–methanol. After vortexing, shaking, and centrifugation, supernatants were combined and concentrated under nitrogen and then redissolved in 5% aqueous methanol. The solution was purified with Oasis WAX solid-phase extraction cartridges, reconcentrated, filtered, and mixed with 2 ng of a ^13^C-labeled internal standard. For vegetable samples, 0.5 g of dry tissue underwent a similar extraction process with the addition of ENVI-Carb to remove pigments. All procedures were conducted without fluorinated consumables to avoid contamination. Quality control parameters, including recovery rates and repeatability, are provided in Text S2.

Quantification of PFAS was performed using an Agilent 1290/6470 high-performance liquid chromatography coupled with tandem mass spectrometry (HPLC–MS/MS) system equipped with a Waters BEH C18 column (2.1 mm × 50 mm, 1.8 μm) maintained at 40 °C. The mobile phase consisted of 2 mM ammonium acetate in water (A) and methanol (B), delivered at a flow rate of 0.4 mL/min under gradient conditions (Table S4). Detection was conducted using negative electrospray ionization in multiple reaction monitoring (ESI-MRM) mode, with parameters detailed in Table S3. Blanks were analyzed every 10 samples, and the system was rinsed with methanol between runs to minimize cross-contamination. The method demonstrated excellent linearity (0.05–50 μg/L, *R* ^2^ > 0.99), recovery rates between 61 and 112%, relative standard deviations below 15%, and detection/quantification limits defined by signal-to-noise ratios of 3 and 10, respectively (Table S5).

### 2.5 Root Chemical Trait Analysis

Functional group composition of root samples was characterized using solid-state ^13^C nuclear magnetic resonance (^13^C NMR) to determine alkyl (chemical shift of 0–50 ppm), *O*-alkyl (50–110 ppm), aromatic and phenolic (110–160 ppm), and carboxyl and carbonyl (160–220 ppm) carbon composition (Text S3).^21,22^

For root-derived DOM analyses, root samples were extracted by using a water-based method. Approximately 0.2 g of homogenized root tissue was mixed with ultrapure water at a 1:80 mass ratio, shaken in the dark for 2 h, and filtered through a 0.45 μm poly­(ether sulfone) membrane. Dissolved organic matter (DOM) concentration in filtered solution was measured as dissolved organic carbon (DOC) by using a TOC analyzer after removing inorganic carbon. Then, the DOM concentration in roots mg-C/g-root was calculated based on the root-to-water extraction ratio. Absorbance spectrum and fluorescence excitation–emission matrix of filtered solution were obtained by using a Horiba Aqualog spectrofluorometer. Specific ultraviolet absorbance at 254 nm, an indicator of DOM aromaticity, was calculated as the absorbance at 254 nm divided by the DOC concentration. The slope ratio (SR), an indicator that negatively correlated with the DOM molecular size, was calculated as the ratio of the logarithmically transformed absorbance spectrum slope at 275–295 nm to that estimated in the range of 350–400 nm. Two fluorescent DOM components, including humic-like and protein-like components, were determined using excitation–emission matrix with parallel factor analysis (PARAFAC) modeling (Text S4).

### 2.6 Biological Accumulation Factor and Translocation Factor

PFAS bioaccumulation and translocation within plants were quantified using three indices: the root bioaccumulation factor (BAF_rs_), the translocation factor from root to aerial parts (TF_ar_), and the bioaccumulation factor for edible tissues (BAF_es_). These were calculated using eqs 1–3.^9,23^

1 $${\mathrm{BAF}}_{\mathrm{rs}}=\frac{C_{\mathrm{root}}}{C_{\mathrm{soil}}}$$

2 $${\mathrm{TF}}_{\mathrm{ar}}=\frac{C_{\mathrm{aerial}}}{C_{\mathrm{root}}}$$

3 $${\mathrm{BAF}}_{\mathrm{es}}=\frac{C_{\mathrm{edible}}}{C_{\mathrm{soil}}}$$

where *C*_soil_ represents the PFAS content (ng/g) in the rhizosphere soil of vegetables; *C*_root_, *C*_aerial_, and *C*_edible_ represent the PFAS content (ng/g) in the root, aerial, and edible parts of vegetables, respectively.

### 2.7 Health Risk Assessment

The estimated daily intake (EDI) of PFAS resulting from local residents’ consumption of vegetables (ng/(kg d)) and the associated health risks can be calculated using eq 4.^5^

4 $$\mathrm{EDI}=\frac{C_{\mathrm{edible}}\times C_{\mathrm{factor}}\times D_{\mathrm{intake}}}{B_{\mathrm{average},\mathrm{weight}}}$$

where EDI represents the estimated daily intake of PFAS (ng/(kg d)); *C*_edible_ denotes the PFAS concentration in edible part of vegetables (ng/g); *C*_factor_ refers to the conversion factor from fresh weight to dry weight, set at 0.085;^7^ *D*_intake_ represents the average daily vegetable consumption: 345 and 232 g/(person d) for adults and children, respectively;^24^ *B*_average,weight_ represents the average body weight, with 62.7 and 32.7 kg for adults and children, respectively.^7^

## 3 Results and Discussion

### 3.1 PFAS Concentrations in Aerial, Root, and Edible Parts of Vegetables

Seven PFAS congeners were detected in the aerial tissues of 20 vegetable species (Table S6), whereas the remaining target PFASs were below the limits of quantification in most samples and, therefore, are not discussed further. Their concentration profiles showed a clear dependence on PFAS chain length. Short-chain (C4–C7) perfluoroalkyl acids (S-PFAAs: perfluorobutanoic, perfluoropentanoic, perfluorohexanoic, and perfluoroheptanoic acids, i.e., PFBA, PFPeA, PFHxA, and PFHpA, respectively) dominated the PFAS composition, accounting for 89.8–96.0% of the total PFAS. Among them, PFBA (C4) was consistently the most abundant, contributing 70.4–77.1%. As shown in Figure 1, S-PFAA concentrations in the aerial parts of all four vegetable categories were significantly higher than those of their long-chain (C8–C9) homologues (L-PFAAs: perfluorooctanoic acid, perfluorononanoic acid, and perfluorooctanesulfonate, i.e., PFOA, PFNA, and PFOS, respectively). This pattern is attributed to the greater hydrophobicity and larger molecular volumes of L-PFAAs, which increase their affinity for biological macromolecules such as proteins and lipids and reduce their mobility across physiological barriers like the Casparian strip.^5,25,26^ These characteristics collectively hinder their translocation to aboveground tissues, especially when compared to the more mobile S-PFAAs.

**Figure 1:**
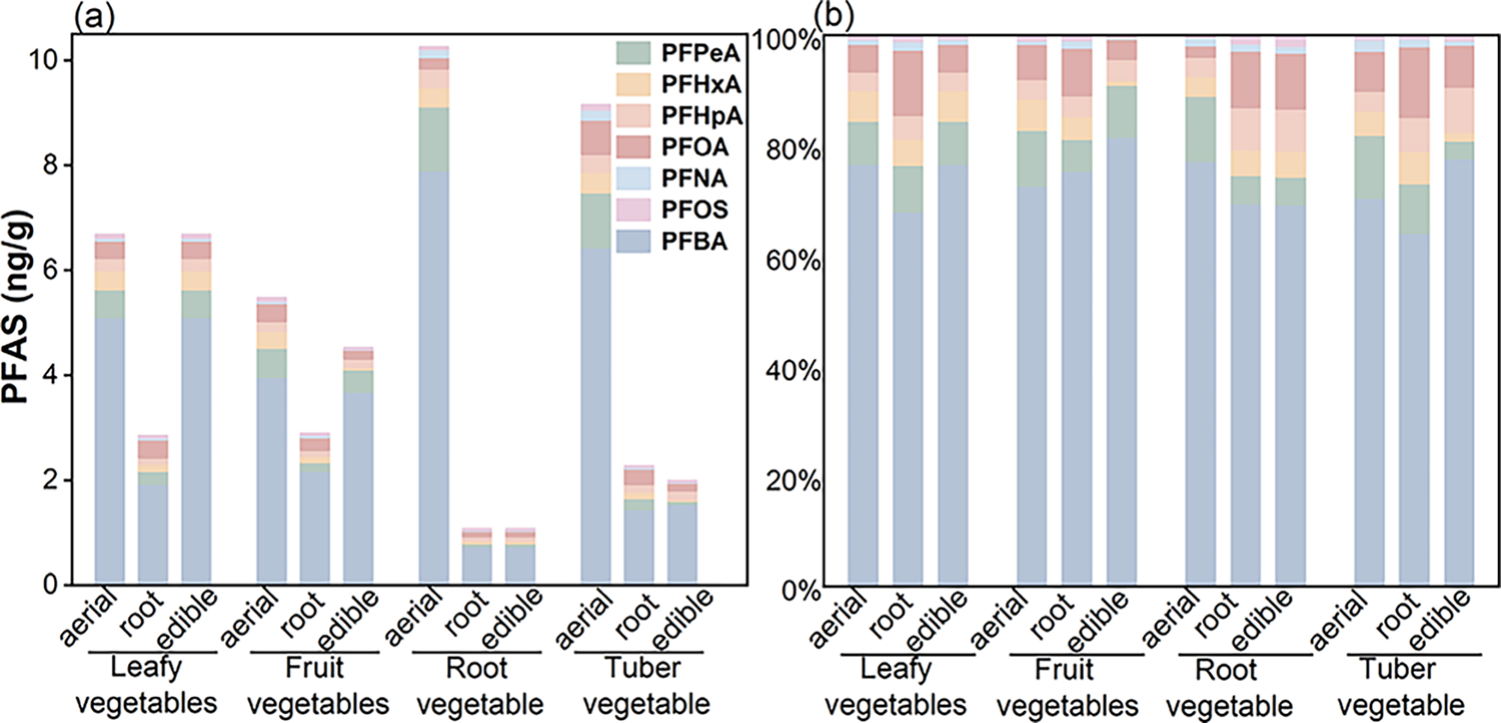
Mean concentrations (ng/g) and compositional profiles of PFAS congeners in aerial, root, and edible parts of the four vegetable categories.

A similar composition was observed in the root tissues, where PFBA (C4) again predominated, representing 67.8–75.3% of total PFAS (Table S6). S-PFAAs (C4–C7) concentrations are 1–2 orders of magnitude higher than L-PFAAs (C8–C9) concentrations in roots (Figure 1), which can be primarily attributed to the decreasing PFAS mobility with increasing carbon chain length.^26,27^ L-PFAAs showed reduced translocation from soil to root tissues compared to that of S-PFAAs. Among vegetable categories, leafy vegetables exhibited the highest level of PFAS accumulation in roots, with a total PFAS concentration (∑PFAS) of 8.92 ng/g (mean). This was significantly higher than the levels observed in tuber vegetable (mean: 6.15 ng/g), root vegetable (mean: 4.78 ng/g), and fruit vegetable (mean: 5.34 ng/g; *p* < 0.05; Figure 1), indicating a stronger enrichment capacity in leafy varieties.

The distribution characteristics of PFAS in edible parts exhibit significant organ specificity (Table S6). PFAS concentrations in the edible parts followed the order: leafy > fruit > tuber > root vegetables. Additionally, the ∑PFAS concentration in leafy vegetables (9.24 ± 1.05 ng/g) was on average 2.4 times higher than that in root vegetables. This disparity may be partly explained by the ability of leafy vegetables to directly absorb atmospheric PFAS via their well-developed stomatal systems, in addition to the dominant contribution from root uptake from soils.^28^ In contrast, the rapid root biomass growth of root vegetables can dilute the PFAS concentrations in their edible tissues. Notably, PFBA remained the dominant congener across all edible tissues, contributing 69.2–81.5% of total PFAS. This is likely because PFBA has a molecular diameter of 0.43 nm, enabling it to penetrate the pore size limitations of the Casparian strip.^5^ Interestingly, root vegetables, which primarily rely on soil-derived PFAS and lack direct atmospheric exposure pathways,^29^ exhibited significantly lower accumulation levels (mean: 3.85 ng/g). These findings highlight the importance of organ-specific uptake mechanisms and their implications for dietary exposure and environmental source tracking. The potential protective role of anatomical barriers in limiting PFAS accumulation, particularly in root vegetables, warrants further investigation.

### 3.2 Accumulation and Translocation Characteristics of PFAS in Aerial, Root, and Edible Parts of Vegetables

This study used TF_ar_ to characterize the transfer capability of PFAS from roots to the aerial parts of plants. The mean TF_ar_ values for PFBA, PFPeA, PFHxA, PFHpA, and PFOA, PFNA, and PFOS in 20 vegetable species were all greater than 1 (Figure 2; Table S7), indicating that PFAS is more likely to be transported to the aerial parts of vegetables. The TF_ar_ values for S-PFAAs were higher than those for L-PFAAs. This finding supports that L-PFAAs were often hindered by biological barriers and retained on root surfaces due to their relatively higher hydrophobicity and larger molecular volume,^9^ while S-PFAAs translocated more extensively to aerial parts.^3,5^

**Figure 2:**
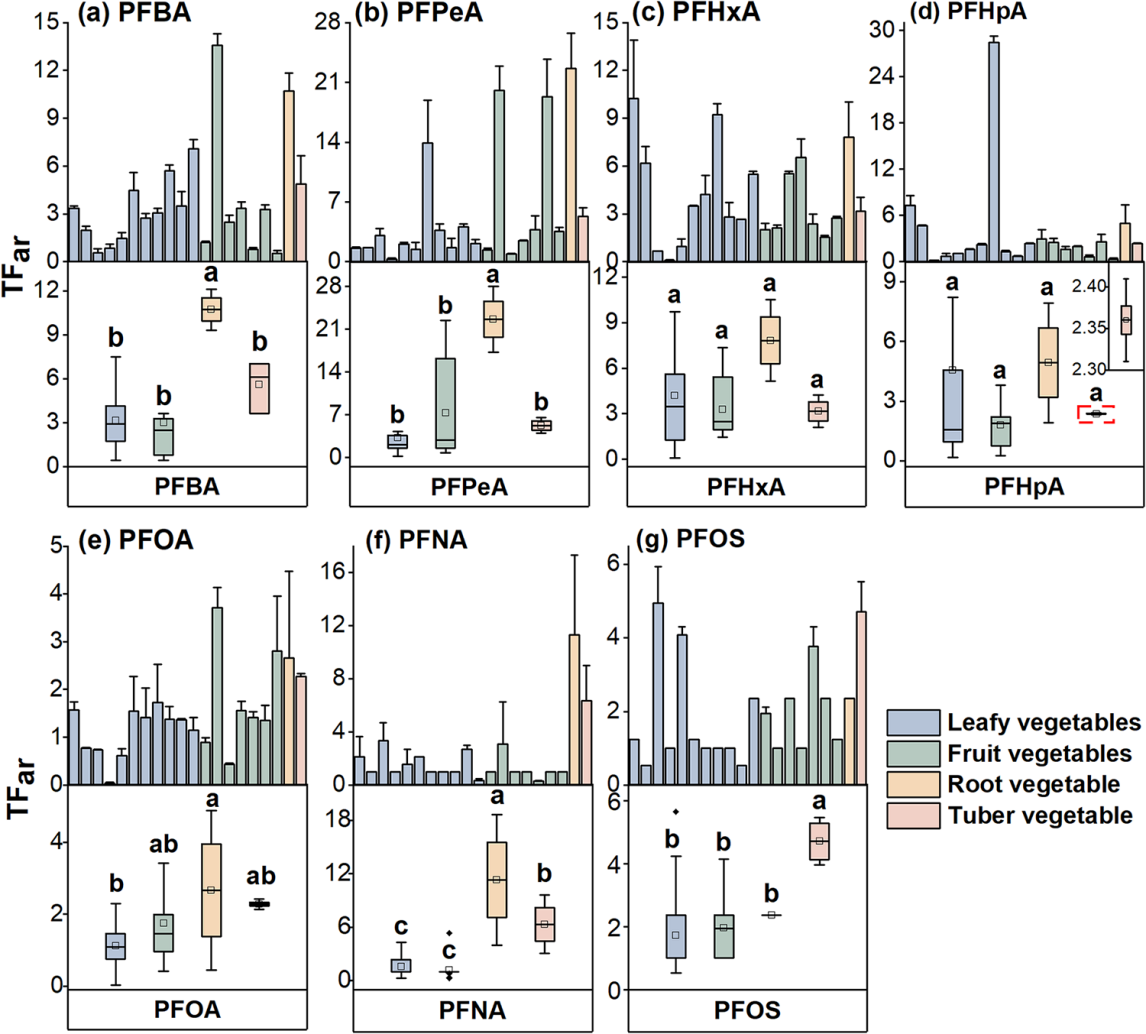
Translocation factors (TF_ar_) of PFBA (a), PFPeA (b), PFHxA (c), PFHpA (d), PFOA (e), PFNA (f), and PFOS (g) from roots to aerial parts in vegetables, illustrating chain-length-dependent differences in mobility. Bar charts show values for each vegetable species (mean ± standard deviation). Box plots show the maximum, 75, 50, 25%, and minimum values for four vegetable categories, with the squared dots showing mean values. Vegetables in the upper panels are ordered by Chinese cabbage, spinach, shallot, Chinese chives, cabbage, celery, Chinese lettuce, garland chrysanthemum, coriander, leafy lettuce, rape (11 leafy vegetables), cucumber, kidney bean, pepper, winged bean, eggplant, tomato, zucchini (7 fruit vegetables), carrot (1 root vegetable), and potato (1 tuber vegetable).

Among the vegetable categories, root vegetables exhibited the highest PFAS translocation capacity. For example, the TF_ar_ values for PFBA, PFPeA, and PFOA in root vegetables reached 10.72 ± 1.76, 22.62 ± 1.06, and 2.66 ± 0.11, respectively, significantly higher than those in leafy vegetables (*p* < 0.05). This result may be because carrots, a representative root vegetable, have less developed Casparian strip structures within roots compared to other species,^25^ thereby allowing both S-PFAAs and L-PFAAs to move freely via the apoplastic pathway and accumulate in aerial parts.^26^ Within leafy vegetables, rape had the highest TF_ar_ value and shallot had the lowest one; within fruit vegetables, kidney bean had the highest TF_ar_ value, and zucchini had the lowest one.

BAF_rs_ results indicate that the bioaccumulation capacity of PFAS from soils to vegetable root systems also exhibited a chain-length dependency (Figure 3). On average, BAF_rs_ were 5.3 times higher for S-PFAAs (2.89 ± 1.05) than for L-PFAAs (0.55 ± 0.17). This difference can be explained by competition for sorption sites between PFAS of varying chain lengths and the strong affinity of L-PFAAs for soil organic matter, which enhances their retention in the soil matrix.^26^ Long-term sewage irrigation may also introduce heavy metals that influence PFAS behavior through competitive adsorption or synergistic transport via plant roots.^26^ While the present study focused on PFAS dynamics, future work should elucidate the coupled interactions between PFAS and heavy metals in the soil–vegetable continuum.^30^ The relatively higher log*K*ow values of L-PFAAs^26^ further reduce their bioavailability and plant uptake efficiency.^3,5^ Furthermore, the BAF_rs_ values generally followed the following order: PFBA > PFPeA > PFHxA > PFHp > PFOA ≈ PFNA. This gradient reflects the superior water solubility, lower soil sorption affinity, and smaller molecular sizes of S-PFAAs, which facilitate their transport from soil to plant roots.^31^ Conversely, BAFrs values for L-PFAAs were generally below 1 (Figure 3), indicating limited root uptake and reinforcing the role of carbon chain length as a key regulator of PFAS bioaccumulation.^25,32^

**Figure 3:**
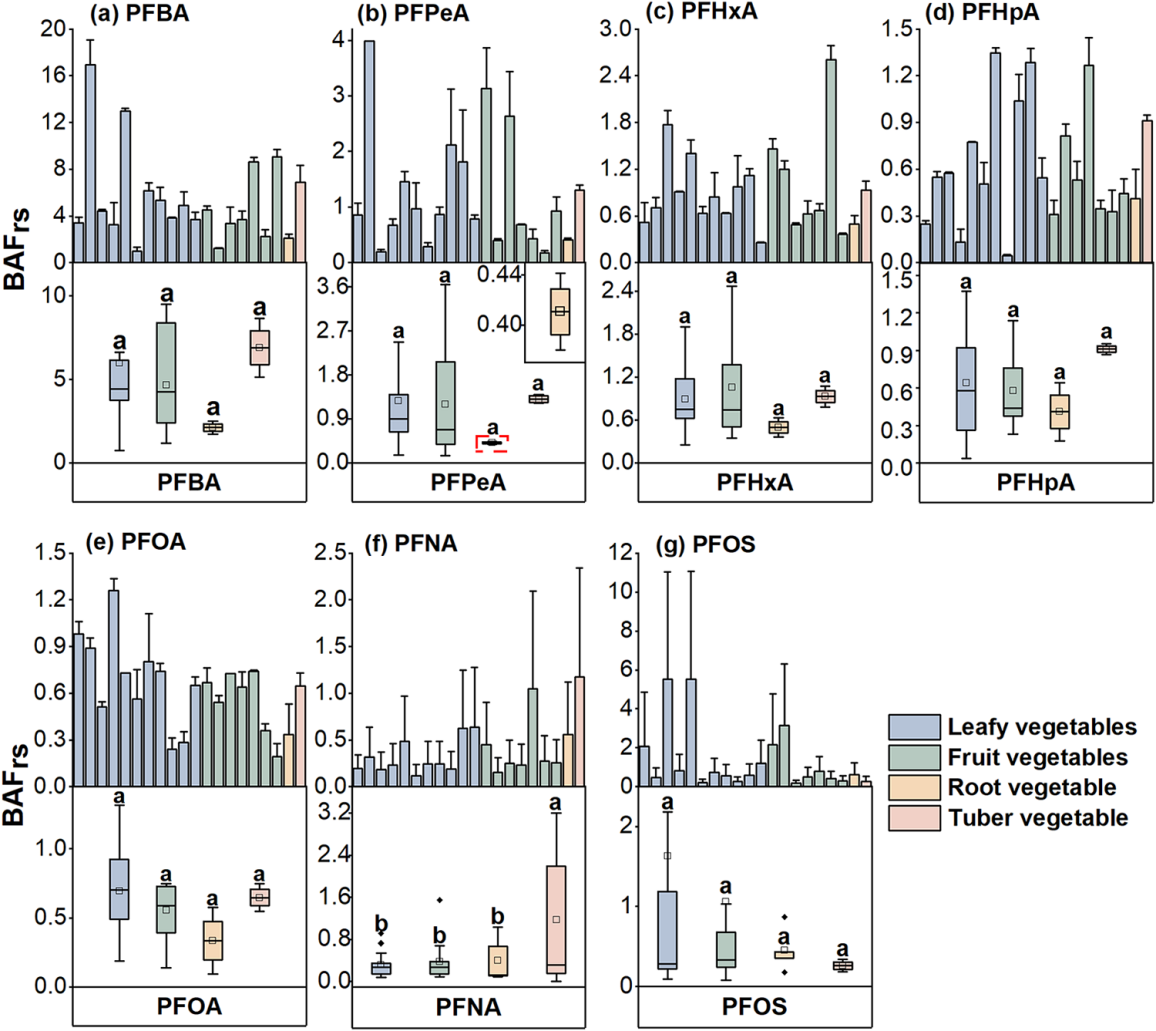
Bioaccumulation factors (BAF_rs_) of PFBA (a), PFPeA (b), PFHxA (c), PFHpA (d), PFOA (e), PFNA (f), and PFOS (g) in root parts of vegetables from soils. Bar charts show values for each vegetable species (mean ± standard deviation). Box plots show the maximum, 75, 50, 25%, and minimum values for four vegetable categories, with the squared dots showing mean values. Vegetables in the upper panels are ranked in the same order as those in Figure 2.

Bioaccumulation in edible plant parts, expressed as BAF_es_, also revealed distinct vegetable- and organ-specific patterns (Figure 4). Among all vegetable categories, leafy vegetables exhibited the highest bioaccumulation capacity for S-PFAAs in edible parts, with PFBA (C4) having a BAF_es_ value of 15.54. PFBA consistently exhibited the highest BAF_es_ among all of the PFAS congeners. Its values were 4–50 times greater than those of its homologues, likely due to its unique molecular characteristics. The short carbon chain and high solubility^33^ allow PFBA to bypass structural barriers such as the Casparian strip and efficiently translocate via the transpiration stream to edible tissues.^5^ The BAF_es_ of PFHxA was significantly higher in leafy vegetables than in fruit, root, and tuber vegetables (*p* < 0.05). Such differences can be partially caused by the dual exposure pathways in leafy vegetables: in addition to root-mediated uptake,^5^ the aerial portions of these plants can directly adsorb atmospheric PFAS through their stomatal systems.^28^ In contrast, tuber and root vegetables rely predominantly on root-mediated transport due to their subterranean edible parts, limiting their exposure to airborne contaminants. Within leafy vegetables, spinach and rape had the highest BAF_es_ values, while Chinese chives and cabbage had the lowest ones; within fruit vegetables, pepper and eggplant had the highest BAF_es_ values, while cucumber and tomato had the lowest ones. Note that Chinese chives, cabbage, cucumber, and tomato had BAF_es_ comparable to carrot, suggesting that they may also cause less risk of PFAS exposure via edible part consumption.

**Figure 4:**
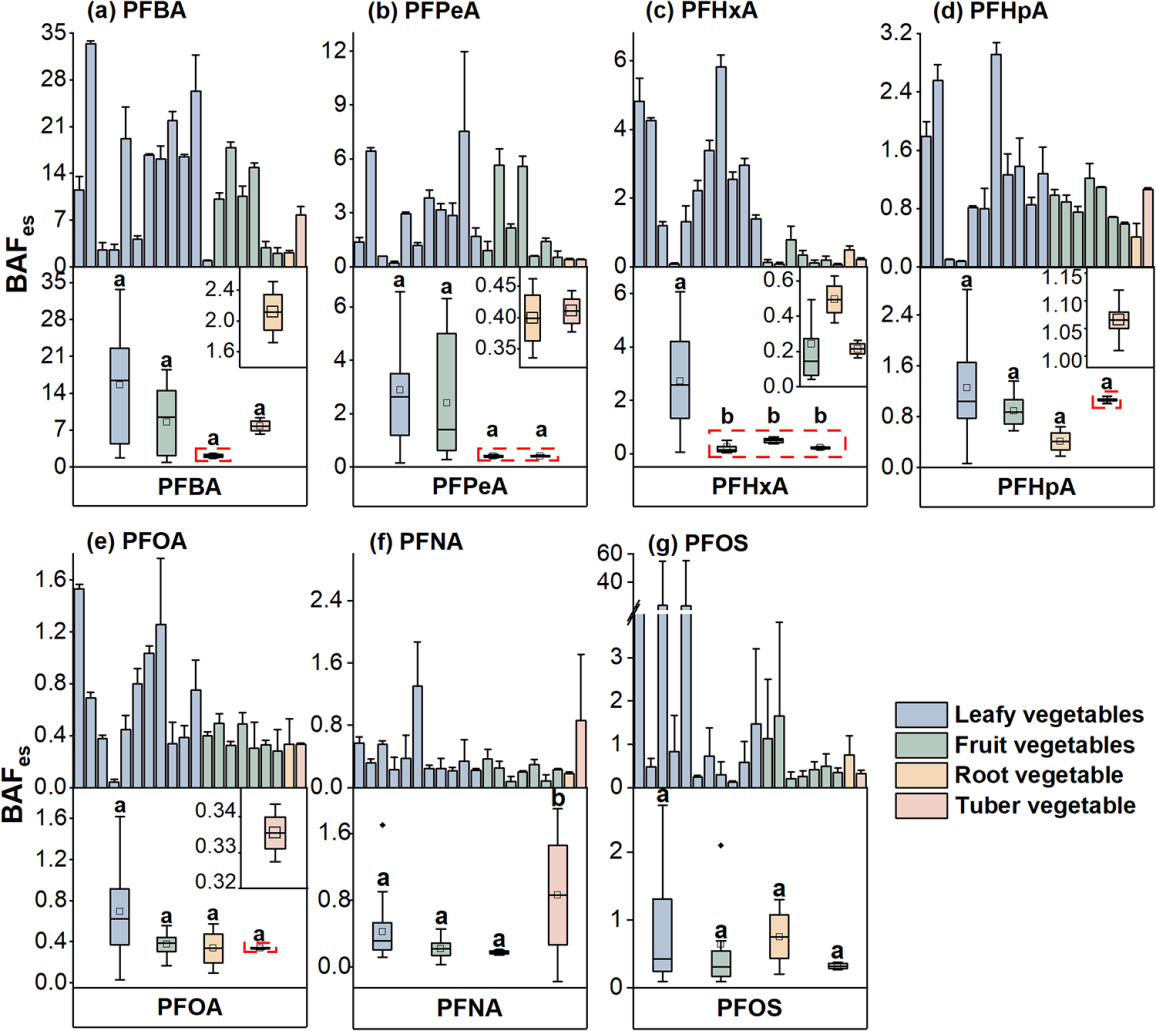
Bioaccumulation factors (BAF_es_) of PFBA (a), PFPeA (b), PFHxA (c), PFHpA (d), PFOA (e), PFNA (f), and PFOS (g) in the edible parts of vegetables from soils. Bar charts show values for each vegetable species (mean ± standard deviation). Box plots show the maximum, 75, 50, 25%, and minimum values for four vegetable categories, with the squared dots showing mean values. Vegetables in the upper panels are ranked in the same order as those in Figure 2.

### 3.3 Regulatory Effects of Root Chemical Traits on PFAS Accumulation

Solid-state ^13^C NMR spectroscopy was employed to elucidate the molecular structural characteristics of vegetable roots (Figure S2). Alkyl carbon, primarily associated with lipids, accounted for 13.8 ± 2.8% of the signal. *O*-alkyl carbon, originating mainly from carbohydrates such as cellulose and hemicellulose, dominated the spectra with relative abundances of 70.8 ± 4.7%. Aromatic and phenolic carbon, indicating lignin-derived compounds, comprised 7.7 ± 2.7%. Finally, carboxyl and carbonyl carbon, reflective of organic acids and quinones, contributed 7.7 ± 1.4%. Although no statistically significant differences were found in the distribution of functional groups across the four vegetable categories (*p* > 0.05), the tuber vegetable potato exhibited a notably higher proportion of aromatic carbon (11.4%) compared with the other types. This high aromatic carbon content may relate to specific biological adaptations, such as high phenylpropanoid metabolism, which supports stress tolerance and environmental resilience.^10,34,36–38^

Root-derived dissolved organic matter (DOM) showed significant interspecific variability (Figure S3). Root and leafy vegetables exhibited DOM contents 6–7.5 times greater than those of fruit vegetables. Carrots (root vegetable) showed exceptionally high DOM content (149.68 ± 16.70 mg-C/g-root), surpassing that of potatoes (tuber vegetable) by over 9-fold. Leeks, a leafy vegetable, showed the highest DOM flux overall (254.22 ± 17.95 mg of C/g of root), whereas eggplant, a fruit vegetable, exhibited the lowest (8.86 ± 0.56 mg-C/g-root). Leafy vegetables had lower SUVA_254_ values and higher SR values than fruit vegetables, indicating that their root-derived DOM contains more low-molecular-weight and less aromatic components. These differences likely reflect distinct carbon allocation strategies: fruit vegetables prioritize reproductive growth, limiting soluble carbon release through roots, while simpler, fast-growing leafy vegetables contain greater quantities of labile and low-molecular-weight DOM.^39^

To further characterize root-derived DOM, we employed EEM spectroscopy coupled with PARAFAC, revealing notable compositional differences among vegetable categories (Figure S4). Root vegetables exhibited significantly higher proportions of humic-like C1 (60.6 ± 2.7%) compared to leafy (37.8 ± 8.1%), fruit (29.1 ± 10.2%), and tuber vegetables (31.8 ± 4.3%) (*p* < 0.05). This humic-like signature is linked to the polymerization of secondary metabolites in root tissues.^35^ Conversely, the root vegetable had a significantly lower proportion of the protein-like component C2 than other vegetable categories.

Pearson correlation analysis revealed that specific chemical traits of root organic matter were significantly linked to root PFAS accumulation (Figures 5 and S5). PFBA concentration in roots was significantly positively correlated with the relative abundance of alkyl C across vegetable species (*p* < 0.01), likely due to the hydrophobic interaction between the lipophilic alkyl chain and the perfluoroalkyl tail of PFBA.^26^ ^408^ PFBA and PFPeA concentration in roots was significantly negatively correlated with the relative abundance of *O*-alkyl C (*p* < 0.05), suggesting that carbohydrate components may inhibit their accumulation by competing for adsorption sites.^40^ Furthermore, correlation analysis revealed that root chemical traits were also linked to PFAS translocation and bioaccumulation factors (Figure S6). For instance, BAF_rs_ of PFBA showed a significant positive correlation with the relative abundance of alkyl carbon (*p* < 0.01), suggesting that root lipid content may promote PFBA accumulation in roots. Conversely, BAF_rs_ of PFBA exhibited a negative correlation with O-alkyl carbon abundance (*p* < 0.01). These relationships further underscore the regulatory role of root chemistry in PFAS dynamics within plants.

**Figure 5:**
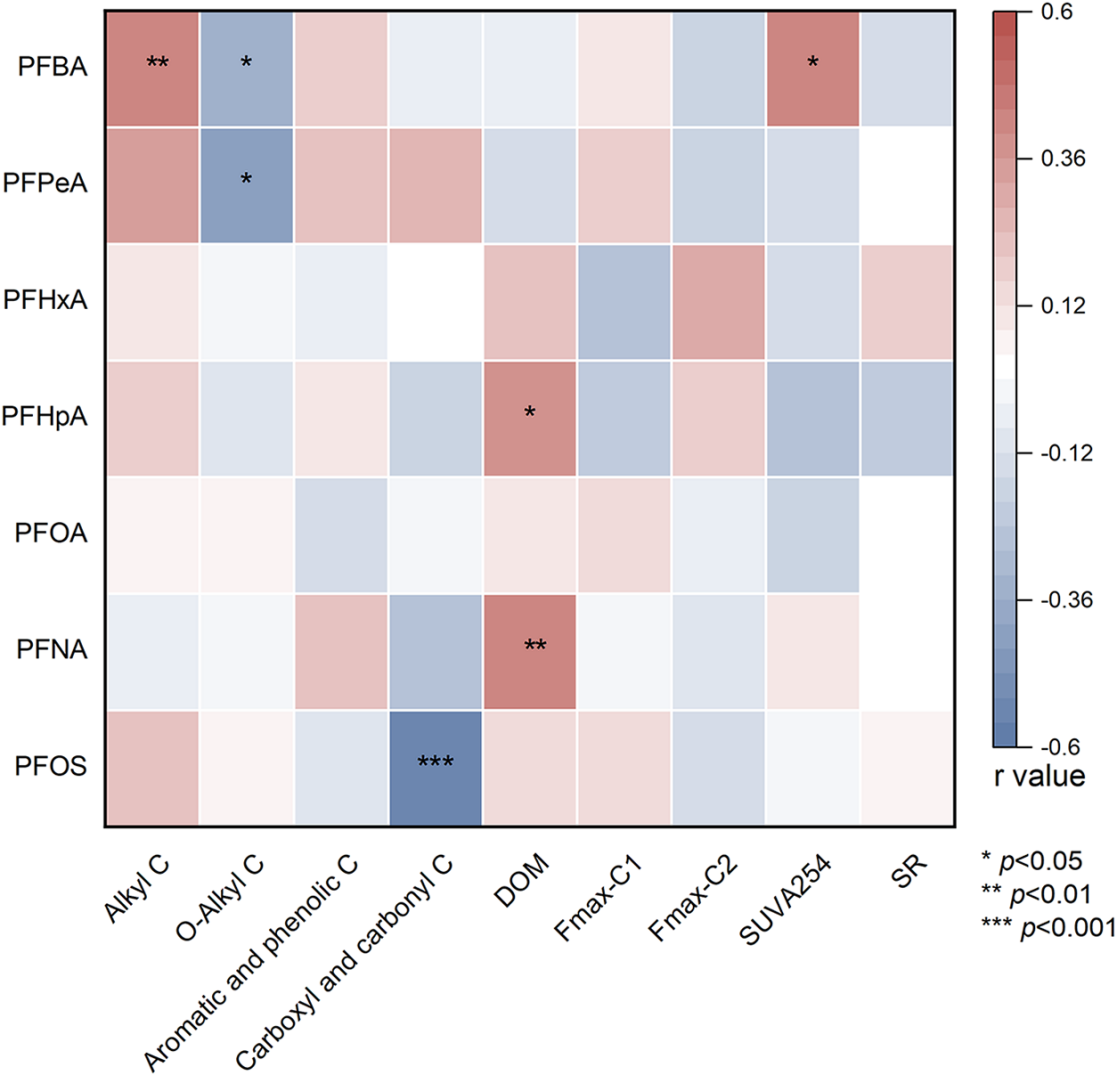
Heatmap showing Pearson correlations between root PFAS concentrations and root chemical traits. DOM: dissolved organic matter content in root indicated by dissolved organic carbon; Fmax: maximum fluorescence for a specific dissolved organic matter component; SUVA254: specific ultraviolet absorbance at 254 nm; SR: slope ratio of absorbance.

Moreover, PFHpA and PFNA concentrations in roots positively correlated with DOM content in roots (*p* < 0.05; *p* < 0.01), indicating that the DOM derived from the root system may enhance the pollutant bioavailability.^10,37^ For example, leafy vegetables, which had the highest average DOM content (101.72 mg of C/g of root), also exhibited significantly higher root concentrations of PFHpA (0.64 ± 0.12 ng/g) and PFNA (0.12 ± 0.03 ng/g) compared to fruit vegetables (22.43 mg of C/g of root; PFHpA: 0.58 ± 0.09 ng/g; PFNA: 0.08 ± 0.02 ng/g). Chinese chives, in particular, showed the strongest DOM secretion and the highest PFNA root concentration (0.19 ± 0.04 ng/g), reinforcing the link between DOM availability and PFAS uptake. Furthermore, the concentration of PFBA in roots was significantly positively correlated with SUVA_254_ (*p* < 0.05), indicating that the content or characteristics of aromatic DOM in roots may be an important factor influencing the accumulation of PFBA in roots. Such result aligns with previous findings that fulvic-like DOM could enhance PFOS sorption through electrostatic interactions.^41^ Overall, our findings suggest that PFAS bioaccumulation is modulated by compound-specific interactions at the root–soil interface, governed by the physicochemical characteristics of root-derived organic matter and the amphiphilic nature of the PFAS molecules. In addition to the root chemical traits investigated herein, root morphological characteristics (e.g., root system architecture, root hair density, and specific root length) are also likely to exert important effects on PFAS accumulation by influencing the root–soil contact area and the exploration of soil volume.^24^ Future studies integrating both chemical and morphological traits would provide a more holistic understanding of interspecific variability in PFAS uptake.

### 3.4 Exposure Risk of PFAS through Vegetable Consumption

Dietary exposure to PFAS via vegetables varied significantly across populations, with children experiencing higher levels of exposure than adults. Based on eq 4, the EDI values for both total PFAS and individual compounds across 20 vegetable species were consistently greater for children (4.03 ng/(kg d)) than for adults (3.12 ng/(kg d)) (*p* < 0.05). This difference primarily reflects children’s higher dietary intake per unit body weight (0.232 kg/d), a parameter commonly used in exposure assessments. These findings are consistent with results from multiple multicenter studies conducted both within China and internationally.^42–44^ The exposure profile showed that S-PFAAs (C4–C7) accounted for 86.4–94.7% of the total EDI (Figure 6). Among these, PFBA was the primary contributor (54.8–93.7% of total EDI), with adult and child EDIs of 1.97 and 2.54 ng/(kg d), respectively, significantly higher than those in Shouguang City, Shandong Province, China (adults: 1.33, children: 2.01 ng/(kg·d)).^45^ EDI values followed the order: leafy vegetables (adults: 3.12; children: 4.03 ng/(kg d)) > fruit vegetables (2.12, 2.73) > tuber vegetables (0.93, 1.20) > root vegetables (0.50, 0.65). The high risk associated with leafy vegetables can be attributed to their higher average bioaccumulation factor (average BAF_es_ = 15.54) and their greater consumption frequency among residents. Among 20 vegetable species, leafy lettuce (PFBA-EDI = 3.97, 5.11), garland chrysanthemum (PFHxA-EDI = 0.35, 0.45), and spinach (PFHpA-EDI = 0.24, 0.31), all belonging to leafy vegetables, exhibited the highest exposure risks. These EDI values were 6–9 times greater than those of the root vegetable (carrot). Notably, the leafy vegetable cabbage, fruit vegetables cucumber and zucchini, similar to the root vegetable carrot, showed the lowest EDI values and thus least risks. Planting these vegetables instead of leafy lettuce could decrease the EDI by up to 90%.

**Figure 6:**
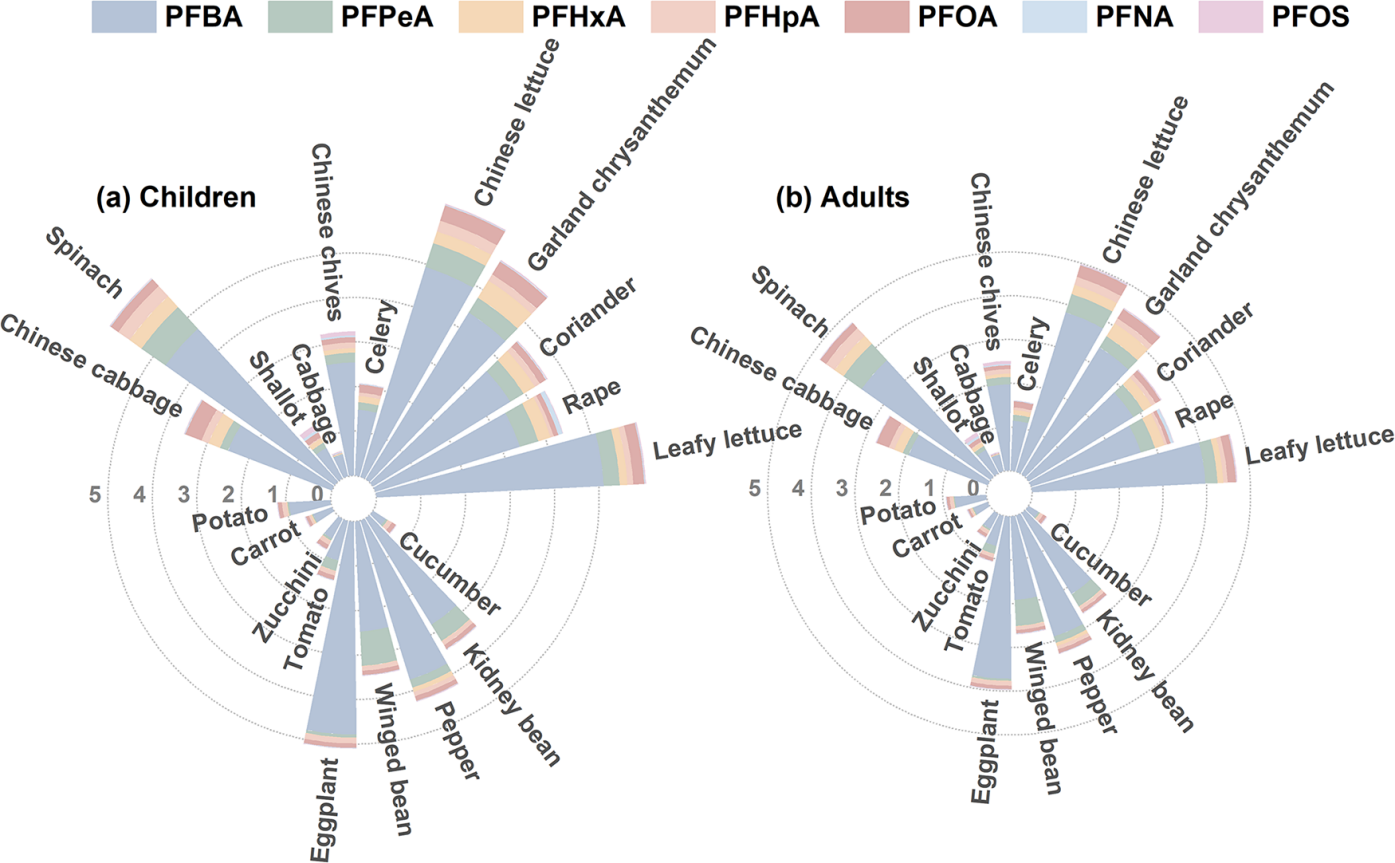
Estimated daily intakes (EDIs) of PFAS from vegetable consumption among residents (ng/(kg d)).

Because no tolerable daily intake (TDI) for short-chain PFAS has been established, only that of PFOA was evaluated. As shown in Table S8, the highest EDI of PFOA from vegetable consumption among local adult residents was 0.30 ng/(kg d) (0.39 ng/(kg d) for children), which is only 0.56–3.92% of the median dietary exposure estimates in European countries (e.g., Belgium: 53.30; Czech Republic: 8.57; Italy: 21.30; Norway: 11.80 ng/(kg d)).^43^ The PFOA exposure levels through vegetable consumption in the study area were significantly lower than those reported internationally. Although China has not yet established dietary guidance values for PFAS, referencing international authoritative standards is an alternative approach. The maximum PFOA-EDI value observed in this study represented only 3.5% of the European Food Safety Authority’s TDI (6 ng/(kg d))^46^ and was even 0.24 and 0.39% of the daily allowable intake levels of the Australian and New Zealand Food Standards Agency (160 ng/(kg d))^47^ and the Danish Environmental Protection Agency (100 ng/(kg d)),^48^ respectively. These comparisons indicate that vegetable-derived PFOA exposure does not pose a remarkable health risk for residents of the study area. Nevertheless, the persistence, bioaccumulation, and resistance to degradation characteristics of PFOA suggest the need for continued monitoring. It is worth noting that cooking processes (e.g., boiling or steaming) may alter PFAS concentrations in vegetables through leaching or thermal degradation, whose magnitude remains uncertain. Future studies should evaluate the influence of common food preparation methods on PFAS levels in order to refine dietary exposure assessments.

## 4 Conclusion

Given the persistence and toxicity of PFAS and their potential risks to human health, particularly through dietary intake, it is essential to understand their accumulation mechanisms in vegetables. This study demonstrated that leafy vegetables had a higher chance to accumulate high PFAS levels, with short-chain PFBA identified as the dominant contaminant. This study further revealed that both root structural composition and DOM may play roles in regulating PFAS accumulation. Specifically, alkyl carbon facilitates PFBA uptake, while *O*-alkyl carbon inhibits it. High DOM concentrations derived from roots promote the uptake of long-chain PFAS (e.g., PFNA) probably through solubilization effects. Children face higher dietary exposure risks from consuming leafy vegetables than adults. To mitigate these risks, we propose to select low-accumulation varieties (e.g., carrot, cabbage, cucumber, and zucchini) instead of high-accumulation varieties (e.g., Chinese lettuce, leafy lettuce, and spinach) and implement multimedia monitoring in polluted farmlands. Moreover, further research is warranted to elucidate the interactions between specific root chemical composition and diverse PFAS compounds, to support multimedia pollutant migration models, and to evaluate the long-term health effects of chronic low-dose exposure as part of improved risk management strategies.

## Supplementary Material



## Notes

Experimental details, materials, and methods, including experimental site and photographs of experimental setup; root chemical trait results based on NMR and fluorescence analyses; and additional results linking root chemical traits and PFAS parameters (PDF)
