# Supplementary material for: Linking Vegetable Per- and Polyfluoroalkyl Substance Accumulation with Root Chemical Traits
Source: ACS Environ Au. 2025 Dec 8;6(1):105–15. doi: 10.1021/acsenvironau.5c00184 (PMC12828611; doi:10.1021/acsenvironau.5c00184)
Supplement: Supplementary file 1 [file vg5c00184_si_001.pdf]

Supporting Information For:

Linking Vegetable Per- and Polyfluoroalkyl Substance Accumulation with Root Chemical Traits

Chun Cao[1,2], Qian Huo[1,2], Qianhui Tang[1,2], Yifan Guo[1,2], Liang Zeng[3,4,5], Yao Cheng[3,4,5], Guomao Zheng[3,4,5], Biwei Yang[3,4,5], Junjian Wang[3,4,5,*]

[1]College of Geography and Environmental Science, Northwest Normal University, Lanzhou, Gansu 730070, China;

[2]Key Laboratory of Resource Environment and Sustainable Development of Oasis, Lanzhou, Gansu 730070, China

[3]State Key Laboratory of Soil Pollution Control and Safety, Southern University of Science and Technology, Shenzhen, Guangdong 518055, China;

[4]Guangdong Provincial Key Laboratory of Soil and Groundwater Pollution Control, School of Environmental Science and Engineering, Southern University of Science and Technology, Shenzhen, Guangdong 518055, China;

[5]State Environmental Protection Key Laboratory of Integrated Surface Water-Groundwater Pollution Control, School of Environmental Science and Engineering, Southern University of Science and Technology, Shenzhen, Guangdong 518055, China;

Corresponding author: Junjian Wang, Email: wangjj@sustech.edu.cn.

CONTENTS

Text S1. Chemical reagents for PFAS extraction, purification and HPLC-MS/MS analysis S4

Text S2. Sample pre-processing workflows for soil and vegetable PFAS extraction S5

Text S3. Experimental protocols for ^{13}C nuclear magnetic resonance (NMR) testing and spectrum data processing. S6

Text S4. Methodological protocols for dissolved organic matter (DOM) fluorescence spectral analysis. S7

Figure S1. Overview map of the experimental site in the study area (a) and the pot experiment setup (b). S8

Figure S2. Relative abundance of functional groups identified by ^{13}C nuclear magnetic resonance spectroscopy in vegetable root tissues. Functional groups include *O*-alkyl carbon (mainly from carbohydrates), alkyl carbon (mainly from lipids), aromatic and phenolic carbon (mainly from lignin), and carboxyl and carbonyl carbon (mainly from organic acids). S9

Figure S3. Variations in dissolved organic matter (DOM) content derived from vegetable root systems. S10

Figure S4. Relative abundances of fluorescence dissolved organic matter components derived from vegetable roots. C1: humic-like component; C2: protein-like component. S11

Figure S5. Scatter plots with regression lines showing Pearson correlations between root PFAS concentrations and root chemical traits. S12

Figure S6. Correlation analysis between root chemical properties and TF and BAF of PFBA. S13

Figure S7. Workflow for soil and vegetable PFAS analyses. S14

Table S1. List of tested plant samples. S15

Table S2. Per- and polyfluoroalkyl substances (PFASs) under investigation. S16

Table S3. List of targeted perfluorinated compounds in the present study and the optimized HPLC-MS/MS parameters. S17

Table S4. HPLC-MS/MS instrument parameters for the quantification of target PFASs...S19

Table S5. LODs, LOQs, MSRs of PFASs in soil and vegetable sample detection. S20
Table S6. Concentration of PFAS in above-ground, below-ground and edible parts of different kinds of vegetables (ng/g). S21
Table S7. BAF_{rs}, TF_{ar}, BAF_{es} of different types of vegetables. S22
Table S8. Estimated daily intakes of PFAS in vegetables ingested by local residents (ng/(kg bw d)). S23

Text S1. Chemical reagents for PFAS extraction, purification and HPLC-MS/MS analysis

Methanol (LC-MS grade) was purchased from Merck, Germany. Ammonium acetate (LC-MS grade) and ammonia (HPLC grade) were purchased from Thermo Fisher Scientific, USA. An Acquity UPLC BEH C18 column and Oasis WAX solid-phase extraction (SPE) columns were obtained from Waters, USA. ENVI-Carb packing powder was purchased from Supelco, USA.

Text S2. Sample pre-processing workflows for soil and vegetable PFAS extraction

Soil PFAS extraction

For soil analysis, 2.0 g of freeze-dried, homogenized soil was weighed into a 50 mL polypropylene centrifuge tube. A 20 µL aliquot of 100 ng/mL surrogate standard (SS, spiked with 2 ng) and 4 mL of 1% ammonia–methanol solution were added. The mixture was vortexed for 2 min at 2000 rpm, sonicated at 60 ℃ for 30 min, and then shaken for 1 h. Following centrifugation at 7000 rpm for 10 min, the extraction was repeated twice, and the supernatants were combined. The combined extract was evaporated to dryness under nitrogen and re-dissolved in 10 mL of 5% methanol–water. The SPE column was activated sequentially with 4 mL of 0.1% ammonia–methanol, 4 mL of methanol, and 4 mL of ultrapure water. After sample loading, the column was rinsed with 25 mM ammonium acetate buffer (pH 4) and vacuum-dried for 30 min. Elution was performed using 4 mL of methanol followed by 4 mL of 0.1% methanol–ammonia. The eluate was evaporated to 1 mL under nitrogen, filtered through a 0.22 µm organic-phase membrane, spiked with 20 µL of 100 ng/mL ^{13}C–internal standard (2 ng), and stored at –4 °C until analysis.

Vegetable PFAS extraction

For vegetable samples, 0.5 g dry weight was weighed, spiked with 2 ng SS, and extracted with 4 mL of 1% ammonia–methanol solution. The mixture was vortexed for 2 min, sonicated at 40 ℃ for 30 min, and shaken for 1 h. Centrifugation was performed at 9000 rpm for 15 min, and the extraction was repeated twice. The supernatants were combined and concentrated to 1.5 mL under nitrogen. The concentrate was transferred to a 2 mL centrifuge tube containing 25 mg of ENVI-Carb adsorbent, vortexed for 1 min, and centrifuged at 13000 rpm for 20 min. The subsequent filtration, spiking, and storage procedures were identical to those described for soil samples. A workflow diagram is shown in Figure S6.

Text S3. Experimental protocols for ^{13}C nuclear magnetic resonance (NMR) testing and spectrum data processing

Dried samples were packed into a 4 mm CP–MAS probe for solid-state ^{13}C NMR analysis.[1] Measurements were conducted at a resonance frequency of 150.9 MHz, with a spinning speed of 12 kHz, a contact time of 4 ms, and a recycle delay of 2 s. After Fourier transformation, spectra were baseline- and phase-corrected. Integration was performed across the following chemical shift regions: 0–50 ppm for alkyl C, 50–110 ppm for *O*-alkyl C, 110–165 ppm for aromatic/phenolic C, and 165–210 ppm for carboxy/carbonyl C. The percentage contribution of each region to the total signal intensity was then calculated.

Text S4. Methodological protocols for dissolved organic matter (DOM) fluorescence spectral analysis

Fluorescence excitation–emission matrix (EEM) spectra were recorded with excitation wavelengths from 240 to 550 nm at 2 nm intervals and emission wavelengths from 245 to 826 nm at ~1 nm intervals, with an integration time of 3 s.[2] Data analysis was performed using the PARAFAC model on the Matlab platform with the drEEM data package to decompose the three-dimensional fluorescence matrix and extract significant components, defined as those validated by three or more independent factors.

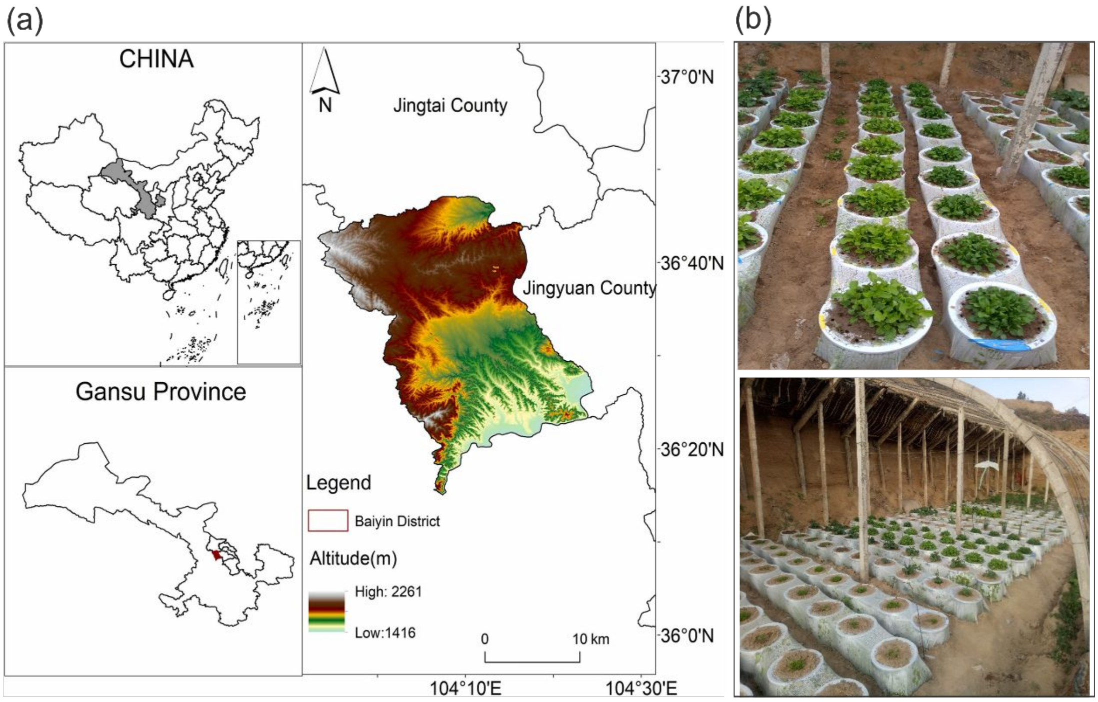

Figure S1. Overview map of the experimental site in the study area (a);[3] the pot experiment setup (b).

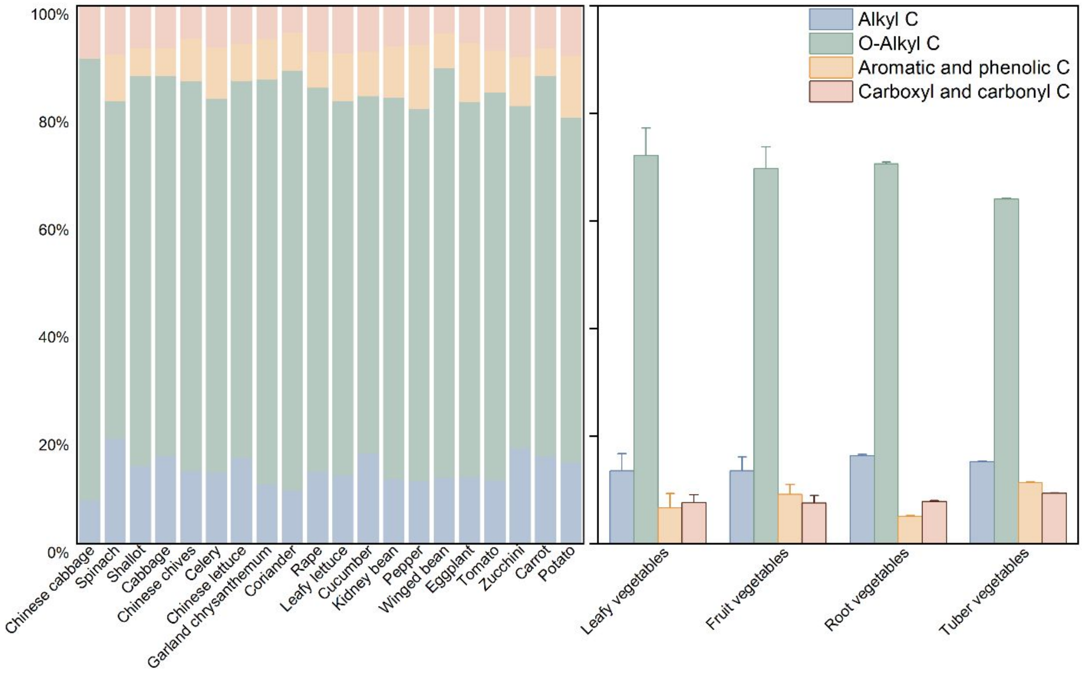

Figure S2. Relative abundance of functional groups identified by ^{13}C nuclear magnetic resonance spectroscopy in vegetable root tissues. Functional groups include *O*-alkyl carbon (mainly from carbohydrates), alkyl carbon (mainly from lipids), aromatic and phenolic carbon (mainly from lignin), and carboxyl and carbonyl carbon (mainly from organic acids).

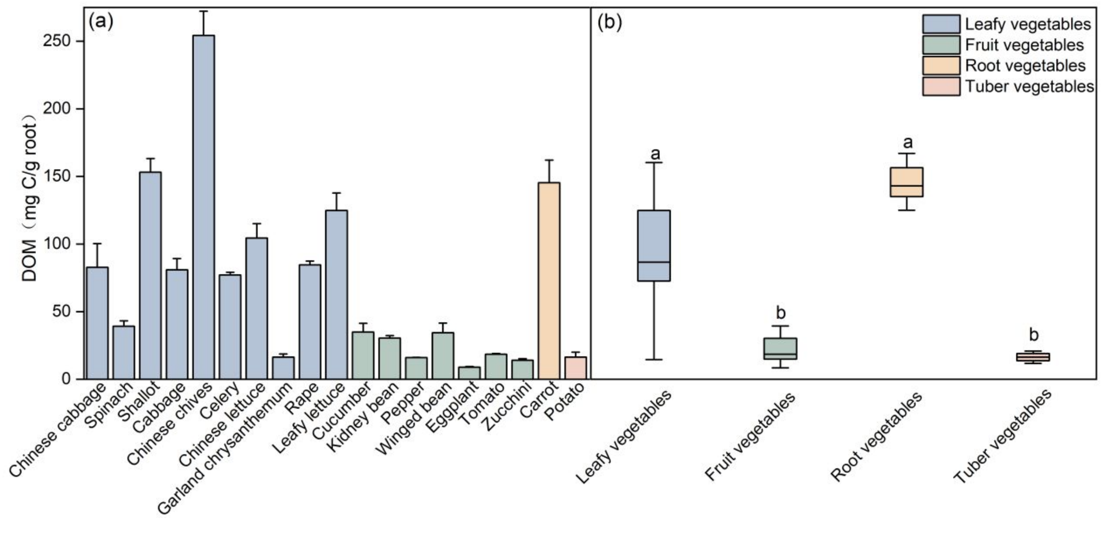

Figure S3. Variations in dissolved organic matter (DOM) content derived from vegetable root systems.

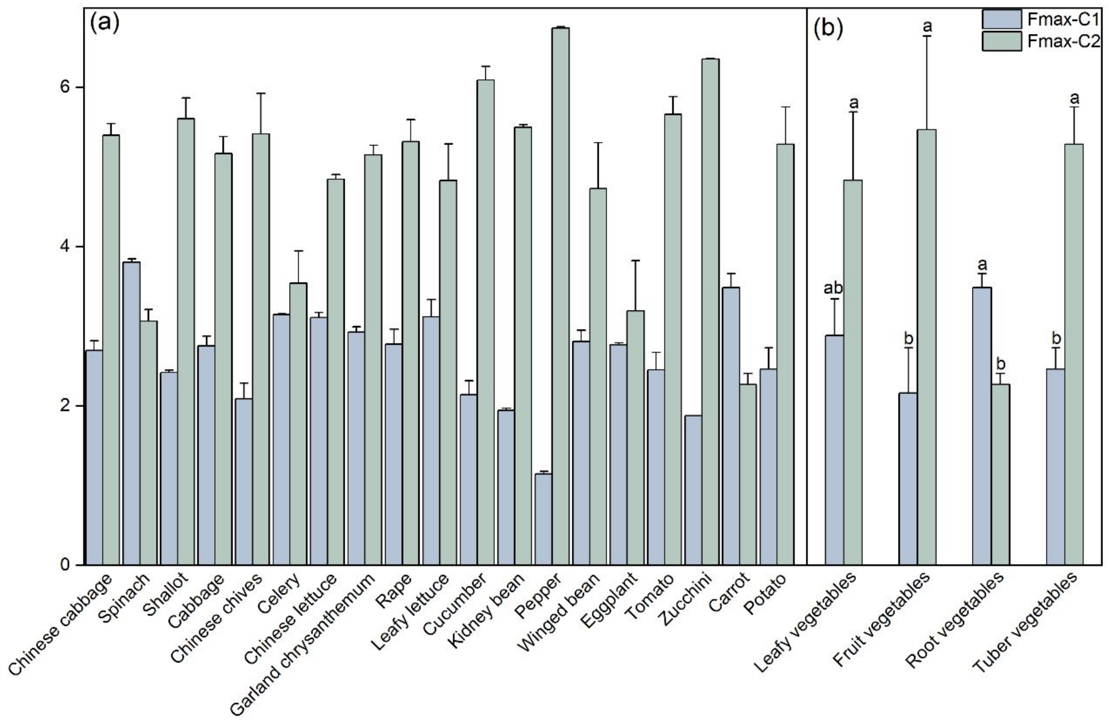

Figure S4. Relative abundances of fluorescence dissolved organic matter components derived from vegetable roots. C1: humic-like component; C2: protein-like component.

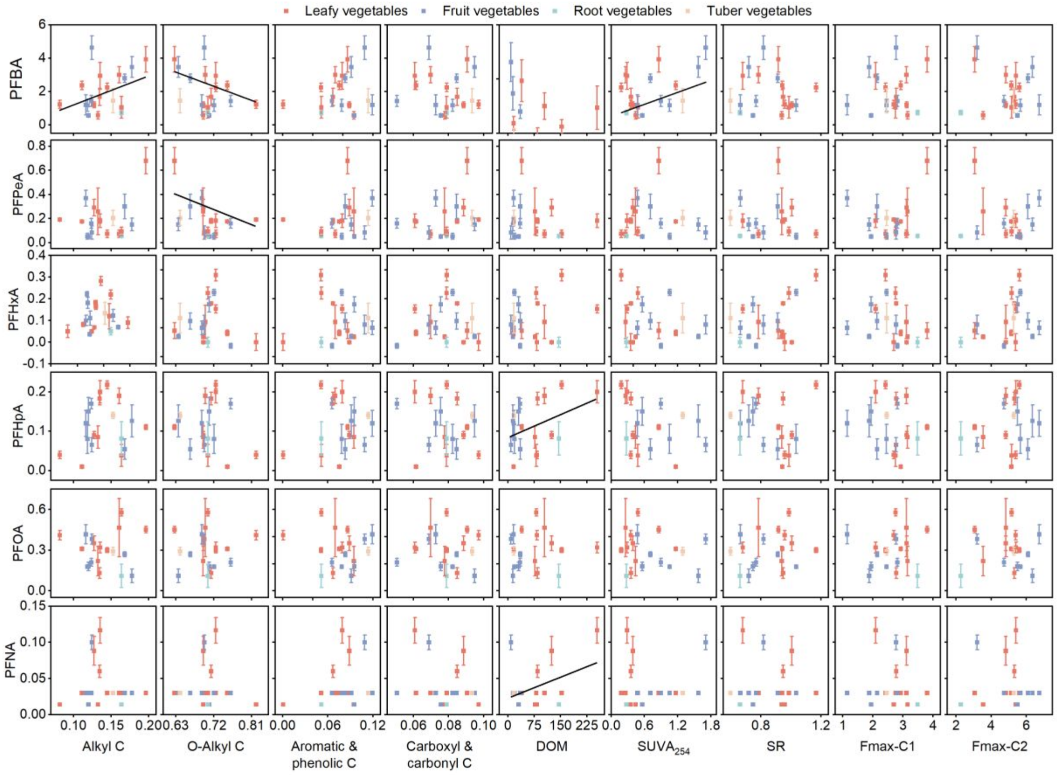

Figure S5. Scatter plots with regression lines showing Pearson correlations between root PFAS concentrations and root chemical traits.

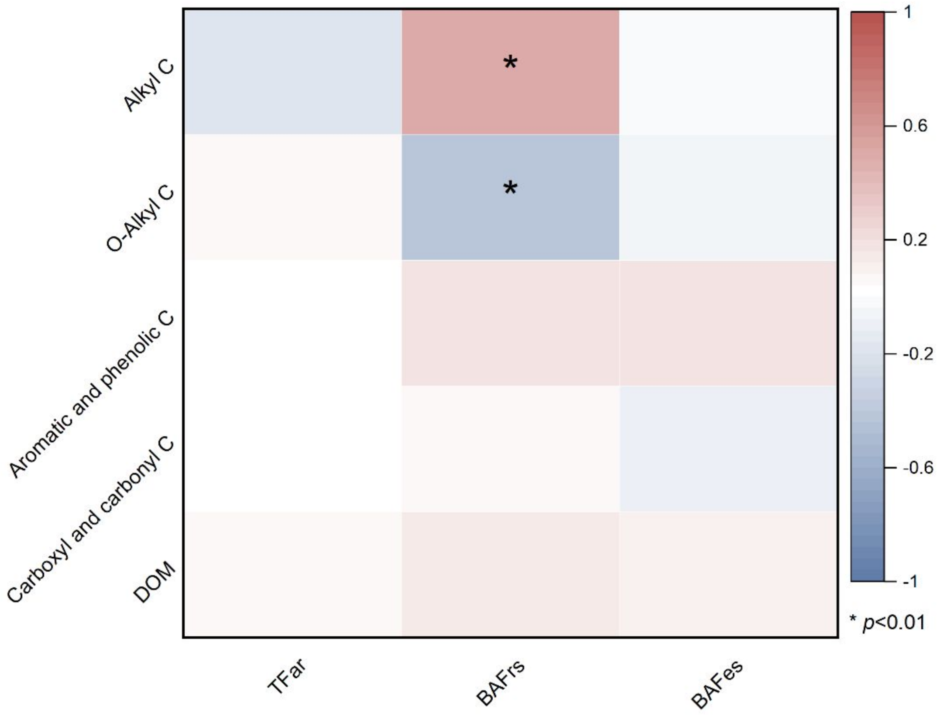

Figure S6. Correlation analysis between root chemical properties and TF and BAF of PFBA.

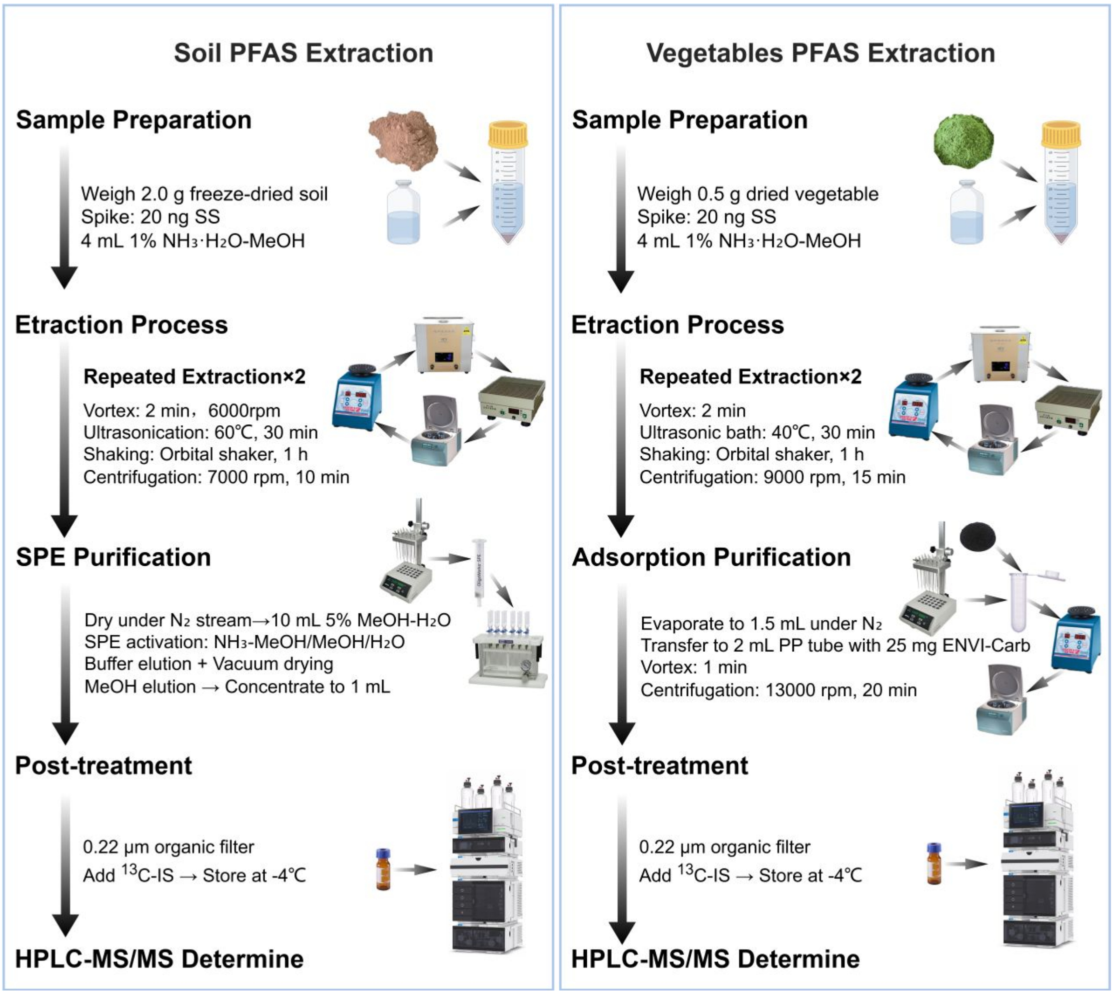

Figure S7. Workflow for soil and vegetable PFAS analyses.

Table S1. List of tested plant samples.

Vegetables	Species in Latin
**Leafy vegetables**	
Chinese cabbage	*Brassica rapa* L. subsp. *pekinensis*
Spinach	*Spinacia oleracea* L.
Shallot	*Allium ascalonicum* L.
Chinese chives	*Allium tuberosum* Rottler ex Spreng
Cabbage	*Brassica oleracea* var. *capitata*
Celery	*Apium graveolens* L.
Chinese lettuce	*Lactuca sativa* var. *ramosa*
Garland chrysanthemum	*Chrysanthemum coronarium* L.
Coriander	*Coriandrum sativum* L.
Leafy lettuce	*Lactuca sativa* var. *longifolia* L.
Rape	*Brassica rapa* L. subsp. *chinensis*
**Fruit vegetables**	
Cucumber	*Cucumis sativus* L.
Kidney bean	*Phaseolus vulgaris* L.
Pepper	*Capsicum annuum* L.
Winged bean	*Vigna unguiculata* (L.) Walp.
Eggplant	*Solanum melongena* L.
Tomato	*Solanum Lycopersicum* L.
Zucchini	*Cucurbita pepo* L.
**Root vegetables**	
Carrot	*Daucus carota* L.
**Tuber vegetables**	
Potato	*Solanum tuberosum* L.

Table S2. Per- and polyfluoroalkyl substances (PFASs) under investigation.

Analyte	Formula	CAS#	Number of Perfluorocarbon (# of CF2)	Molecular Weight (g/mol)
**L-PFAAs**				
PFOA	$C_8HF_{15}O_2$	335-67-1	7	414.07
PFNA	$C_9HF_{17}O_2$	375-95-1	8	464.08
PFDA	$C_{10}HF_{19}O_2$	335-76-2	9	514.08
PFUdA	$C_{11}HF_{21}O_2$	2058-94-8	10	564.09
PFDoA	$C_{12}HF_{23}O_2$	307-55-1	11	614.1
PFTrDA	$C_{13}HF_{25}O_2$	72629-94-8	12	664.1
PFTeDA	$C_{14}HF_{27}O_2$	376-06-7	13	714.11
PFHxDA	$C_{16}HF_{31}O_2$	67905-19-5	15	814.13
PFODA	$C18HF_{35}O_2$	16517-11-6	17	914.1
PFOS	$C_8HF_{17}O_3S$	1763-23-1	8	500.13
PFDS	$C_{10}HF_{21}O_3S$	335-77-3	10	600.15
**S-PFAAs**				
PFBA	$C_4HF_7O_2$	375-22-4	3	214.04
PFPeA	$C_5HF_9O_2$	2706-90-3	4	264.05
PFHxA	$C_6HF_{11}O_2$	307-24-4	5	314.05
PFHpA	$C_7HF_{13}O_2$	375-85-9	6	364.06
PFBS	$C_4HF_9O_3S$	375-73-5	4	300.10
PFHxS	$C_6HF_{13}O_3S$	355-46-4	6	400.12
PFHpS	$C_7HF_{15}O_3S$	375-92-8	7	450.12
**E-PFAS**				
FOSA	$C_8H_2F_{17}NO_2S$	745-91-6	8	499.15
N-MeFOSA	$C_9H_4F_{17}NO_2S$	132445-38-6	8	513.17
N-EtFOSA	$C_{10}H_6F_{17}NO_2S$	4151-50-2	8	527.2
N-MeFOSAA	$C_{11}H_6F_{17}NO_4S$	1400690-70-1	8	574.23
N-EtFOSAA	$C_{10}H_6F_{17}NO_2S$	4151-50-2	6	527.2
FOUEA	$C_{10}H_2F_{16}O_2$	70887-84-2	7	458.1
HFPO-DA	$C_6HF_{11}O_3$	13252-13-6	7	330.05
ADONA	$C_7H_2F_{12}O_4$	919005-14-4	6	378.07
8:2diPAP	$C_{20}H_9F_{34}O_4P$	678-41-1	8	990.2
6:2Cl-PFESA	$C_8HClF_{16}O_4S$	756426-58-1	8	532.58
6:2 FTS	$C_8H_5F_{13}O_3S$	27619-97-2	6	428.17
8:2 FTS	$C_{10}H_5F_{17}O_3S$	39108-34-4	8	528.18

Table S3. List of targeted perfluorinated compounds in the present study and the optimized HPLC-MS/MS parameters.

Analytes	Parent ion - daughter ion (m/z)	Fragmentor (volts)	Collision energy (volts)
PFBA	213-168	64	5
PFPeA	263-218.9/140.8	64	5/15
PFHxA	313-268.9/119	73	5/21
PFHpA	363-319/169	78	5/17
PFOA	413.1-369/169	83	5/17
PFNA	463.1-419/218.9	83	5/17
PFDA	513-468.9/269	93	5/17
PFUdA	563-518.9/268.9	102	5/17
PFDoA	613-569/269	102	9/21
PFTrDA	663.1-619/169	107	9/29
PFTeDA	713.1-668.9/169	112	13/29
PFHxDA	813.1-768.9/168.9	121	13/37
PFODA	912.9-868.9/169.1	121	13/37
FOSA	498-78/48.1	169	37/150
N-MeFOSA	512-169/218.9	160	29/25
N-EtFOSA	526-169/219	165	29/29
N-MeFOSAA	569.7-482.8/418.9	160	10/15
N-EtFOSAA	583.9-482.9/418.8	165	10/15
FOUEA	457-393	83	5
HFPO-DA	285-169	108	12
PFBS	299-80/98.9	149	37/37
PFHxS	399-80/98.9	179	45/41
PFHpS	449-80/98.9	183	49/45
PFOS	499-80/98.9	208	101/49
PFDS	598.9-80/98.9	232	137/53
6:2FTS	427-406.9/79.9	125	24/41
8:2FTS	527-506.8/80.9	170	8/28
9Cl-PF3ONS	530.9-351/83	200	10/101
8:2diPAP	989-543/96.9	130	15/40
NaDONA	376.9-251/85.1	80	5/21
MPFBA (SS)	217-172	64	5
M5PFPeA (SS)	268-223	64	5
M5PFHxA (SS)	318-273	73	5
M4PFHpA (SS)	367-322	78	5
M8PFOA (SS)	421-376	83	5
M9PFNA (SS)	472-427	83	17
M6PFDA (SS)	519-474	93	5
M7PFUdA (SS)	570-525	102	5
MPFDoA (SS)	615-570	102	9
M2PFTeDA (SS)	715-670	112	13
M8FOSA (SS)	506-78	169	37
d3-N-MeFOSAA (SS)	573-419	160	15
d5-N-EtFOSAA (SS)	589-419	165	15
M3PFBS (SS)	302-80	149	37
M3PFHxS (SS)	402-80	179	45

M8PFOS (SS)	507-80	208	101
M2-4:2FTS (SS)	329-81	125	28
M2-6:2FTS (SS)	427-81	125	32
M2-8:2FTS (SS)	529-81	170	36
M3PFBA (IS)	216-172	64	5
M2PFOA (IS)	415-370	83	5
MPFOS (IS)	503-80	208	101

Note: The optimized MRM transitions, fragmentors, and collision energies for surrogate and internal standards of per- and polyfluoroalkyl substances (PFASs) analyzed under ESI mode. IS: internal standard; SS: surrogate standard.

Table S4. HPLC-MS/MS instrument parameters for the quantification of target PFASs.

Instrument Model	Agilent 1290-6470+BEH C18 column (50 × 2.1 mm)			
Column Temperature	40℃			
Mobile phase	2 mM ammonium acetate aqueous solution (A);<br>2 mM ammonium acetate methanol solution (B)			
gradient	Time (min)	Flow Rate(mL/min)	A(%)	B(%)
	Initial time	0.4	90	10
	0.5	0.4	90	10
	1.0	0.4	60	40
	17.5	0.4	0	100
	20.0	0.4	0	100
	20.10	0.4	90	10
Mass Spectrometer	Capillary voltage: -2.8 kV			
	Sheath gas temperature: 330°C			
	Sheath gas flow rate: 11 L/min			
	Drying gas temperature: 300°C			
	Drying gas flow rate: 10 L/min			

Table S5. LODs, LOQs, MSRs of PFASs in soil and vegetable sample detection.

Analytes	Soil			Vegetables		
	LOD (ng/g)	LOQ (ng/g)	MSR (%)	LOD (ng/g)	LOQ (ng/g)	MSR (%)
PFBA	0.01	0.04	101±4	0.07	0.23	93±8
PFPeA	0.02	0.05	94±8	0.04	0.11	89±9
PFHxA	0.01	0.03	91±6	0.03	0.10	87±9
PFHpA	0.01	0.04	93±4	0.03	0.08	86±7
PFOA	0.02	0.05	92±6	0.02	0.06	81±5
PFNA	0.01	0.03	88±3	0.02	0.06	77±7
PFDA	0.01	0.04	92±4	0.04	0.12	84±3
PFUdA	0.01	0.02	91±3	0.03	0.11	91±4
PFDoA	0.01	0.04	85±5	0.02	0.05	80±7
PFTrDA	0.02	0.05	79±2	0.01	0.03	72±5
PFTeDA	0.02	0.06	81±4	0.03	0.10	80±6
PFHxDA	0.01	0.04	89±3	0.02	0.07	68±4
PFODA	0.01	0.03	73±3	0.04	0.11	69±2
PFBS	0.01	0.03	75±7	0.05	0.15	69±5
PFHxS	0.02	0.05	74±6	0.03	0.09	67±8
PFHpS	0.01	0.04	69±2	0.04	0.12	61±4
PFOS	0.01	0.03	89±2	0.03	0.08	77±3
PFDS	0.01	0.03	72±5	0.04	0.11	70±3
FOSA	0.02	0.05	65±4	0.02	0.07	76±4
MeFOSA	0.02	0.06	69±3	0.03	0.10	61±5
EtFOSA	0.02	0.06	66±7	0.03	0.08	65±4
MeFOSAA	0.02	0.05	72±2	0.02	0.05	74±4
EtFOSAA	0.01	0.05	70±1	0.02	0.06	82±8
FOUEA	0.01	0.03	91±4	0.02	0.05	74±5
HFPO-DA	0.01	0.02	89±5	0.03	0.08	80±7
6:2FTS	0.02	0.05	107±12	0.03	0.10	112±13
8:2FTS	0.01	0.02	100±7	0.01	0.03	104±9
NaDONA	0.01	0.02	78±2	0.01	0.04	74±4
6:2Cl-PFESA	0.01	0.02	70±7	0.02	0.05	74±3
8:2diPAP	0.01	0.03	86±3	0.01	0.04	82±7

Table S6. Concentration of PFAS in above-ground, below-ground and edible parts of different kinds of vegetables (ng/g).

Vegetables	PFBA	PFPeA	PFHxA	PFHpA	PFOA	PFNA	PFOS
Aerial							
Leafy vegetables	5.11±2.74a	0.53±0.32b	0.37±0.21a	0.22±0.15a	0.34±0.19b	0.05±0.04b	0.06±0.06a
Fruit vegetables	3.97±1.84a	0.55±0.30b	0.31±0.12a	0.19±0.13a	0.35±0.18b	0.03±0.00b	0.06±0.04a
Root vegetables	7.91±0.63a	1.22±0.07a	0.37±0.03a	0.35±0.01a	0.21±0.03b	0.16±0.06a	0.04±0.00a
Tuber vegetables	6.44±0.99a	1.04±0.11a	0.40±0.05a	0.33±0.02a	0.66±0.05a	0.19±0.06a	0.08±0.01a
Root							
Leafy vegetables	1.93±1.05a	0.24±0.19a	0.14±0.08a	0.12±0.07a	0.34±0.13a	0.04±0.03a	0.03±0.01a
Fruit vegetables	2.18±1.48a	0.17±0.12a	0.12±0.06a	0.11±0.04a	0.25±0.11ab	0.04±0.03a	0.03±0.01a
Root vegetables	0.74±0.14a	0.06±0.01a	0.05±0.02a	0.08±0.04a	0.11±0.09b	0.01±0.00a	0.02±0.00a
Tuber vegetables	1.45±0.73a	0.20±0.06a	0.13±0.05a	0.14±0.01a	0.29±0.03ab	0.03±0.00a	0.02±0.00a
Edible							
Leafy vegetables	5.11±2.74a	0.53±0.32a	0.37±0.21a	0.22±0.15a	0.34±0.19a	0.05±0.04a	0.06±0.06a
Fruit vegetables	3.69±2.83ab	0.43±0.44a	0.04±0.06b	0.17±0.02a	0.17±0.01a	0.01±0.00a	0.02±0.00a
Root vegetables	0.74±0.14b	0.06±0.02a	0.05±0.02b	0.08±0.04a	0.11±0.09a	0.01±0.01a	0.02±0.00a
Tuber vegetables	1.55±0.23ab	0.06±0.02a	0.03±0.01b	0.16±0.01a	0.15±0.03a	0.01±0.04a	0.02±0.00a

Different lower-case letters represent significant differences ($p < 0.05$).

Table S7. BAF_{rs}, TF_{ar}, BAF_{es} of different types of vegetables.

Vegetables	PFBA	PFPeA	PFHxA	PFHpA	PFOA
**BAF_{rs}**					
Leafy vegetables	5.99±4.66a	1.27±1.10a	0.89±0.44a	0.64±0.43a	0.70±0.30a
Fruit vegetables	4.68±2.96a	1.20±1.18a	1.06±0.76a	0.58±0.35a	0.55±0.20a
Root vegetables	2.11±0.32a	0.41±0.03a	0.50±0.11a	0.41±0.19a	0.33±0.20a
Tuber vegetables	6.89±1.45a	1.30±0.08a	0.93±0.12a	0.91±0.04a	0.65±0.08a
**TF_{ar}**					
Leafy vegetables	3.17±1.99b	3.23±3.8b	4.18±3.39a	4.57±7.99a	1.12±0.58b
Fruit vegetables	3.59±4.38b	7.35±8.31b	3.26±1.91a	1.8±1.07a	1.74±1.15ab
Root vegetables	10.72±1.76a	22.62±1.06a	7.82±0.88a	4.94±0.04a	2.66±0.11a
Tuber vegetables	5.60±3.43b	5.27±9.32b	3.16±3.24a	2.36±2.63a	2.28±1.7ab
**BAF_{es}**					
Leafy vegetables	15.54±9.80a	2.89±2.48a	2.73±1.71a	1.26±0.89a	0.70±0.45a
Fruit vegetables	8.47±6.42a	2.40±2.20a	0.24±0.27b	0.89±0.23a	0.38±0.12a
Root vegetables	2.11±0.32a	0.40±0.05a	0.50±0.11b	0.41±0.19a	0.33±0.20a
Tuber vegetables	7.77±1.28a	0.41±0.03a	0.21±0.04b	1.06±0.04a	0.34±0.01a

Different lower-case letters represent significant differences ($p < 0.05$).

Table S8. Estimated daily intakes of PFAS in vegetables ingested by local residents (ng/(kg bw d)).

Vegetables		PFBA		PFPeA		PFHxA		PFHpA		PFOA		PFNA		PFOS		∑PFAS	
		Children	Adults	Children	Adults	Children	Adults	Children	Adults	Children	Adults	Children	Adults	Children	Adults	Children	Adults
Leafy vegetables	Chinese cabbage	2.50	1.94	0.19	0.14	0.28	0.21	0.17	0.13	0.39	0.30	0.02	0.01	0.01	0.01	3.55	2.75
	Spinach	4.66	3.61	0.66	0.51	0.33	0.25	0.31	0.24	0.21	0.16	0.02	0.01	0.01	0.01	6.20	4.81
	Shallot	0.78	0.61	0.13	0.10	0.11	0.09	0.02	0.02	0.13	0.10	0.06	0.05	0.12	0.09	1.36	1.06
	Cabbage	0.50	0.39	0.02	0.01	0.01	0.01	0.01	0.01	0.01	0.01	0.02	0.01	0.02	0.02	0.60	0.46
	Chinese chives	2.54	1.97	0.22	0.17	0.10	0.08	0.13	0.10	0.12	0.09	0.04	0.03	0.09	0.07	3.23	2.50
	Celery	1.48	1.14	0.17	0.14	0.14	0.11	0.08	0.06	0.18	0.14	0.02	0.01	0.01	0.01	2.09	1.62
	Chinese lettuce	4.93	3.82	0.56	0.44	0.28	0.22	0.25	0.19	0.36	0.28	0.02	0.01	0.02	0.02	6.42	4.98
	Garland chrysanthemum	4.40	3.41	0.39	0.30	0.45	0.35	0.17	0.13	0.33	0.25	0.02	0.01	0.02	0.02	5.78	4.48
	Coriander	3.62	2.81	0.35	0.27	0.30	0.23	0.13	0.10	0.16	0.13	0.02	0.01	0.02	0.02	4.60	3.57
	Rape	3.39	2.63	0.44	0.34	0.29	0.23	0.07	0.06	0.11	0.08	0.10	0.07	0.01	0.01	4.41	3.42
	Leafy lettuce	5.11	3.97	0.36	0.28	0.17	0.13	0.13	0.10	0.25	0.19	0.02	0.01	0.02	0.02	6.06	4.70
Fruit vegetables	Cucumber	0.34	0.26	0.05	0.04	0.01	0.01	0.10	0.08	0.10	0.08	0.01	0.01	0.01	0.01	0.62	0.48
	Kidney bean	2.81	2.18	0.43	0.33	0.01	0.01	0.10	0.08	0.10	0.08	0.01	0.01	0.01	0.01	3.47	2.69
	Pepper	3.74	2.90	0.19	0.15	0.10	0.08	0.10	0.08	0.11	0.09	0.01	0.01	0.01	0.01	4.27	3.31
	Winged bean	2.47	1.91	0.77	0.60	0.01	0.01	0.10	0.08	0.10	0.08	0.01	0.01	0.01	0.01	3.47	2.69
	Eggplant	4.78	3.71	0.07	0.05	0.01	0.01	0.12	0.10	0.10	0.08	0.01	0.01	0.01	0.01	5.11	3.96
	Tomato	0.91	0.71	0.24	0.18	0.01	0.01	0.10	0.08	0.10	0.08	0.01	0.01	0.01	0.01	1.38	1.07
	Zucchini	0.50	0.39	0.05	0.04	0.01	0.01	0.10	0.08	0.10	0.08	0.01	0.01	0.01	0.01	0.78	0.60
Root vegetables	Carrot	0.45	0.35	0.03	0.03	0.03	0.02	0.05	0.04	0.07	0.05	0.01	0.01	0.01	0.01	0.65	0.50
Tuber vegetables	Potato	0.93	0.72	0.04	0.03	0.02	0.01	0.10	0.08	0.09	0.07	0.01	0.01	0.01	0.01	1.20	0.93
Leafy vegetables		3.08a	2.39a	0.32a	0.25a	0.22a	0.17a	0.13a	0.10a	0.20a	0.16a	0.03a	0.02a	0.03a	0.03a	4.03a	3.12a
Fruit vegetables		2.22a	1.72a	0.26a	0.20a	0.02b	0.02b	0.10a	0.08a	0.10a	0.08a	0.01a	0.01a	0.01a	0.01a	2.73ab	2.12ab
Root vegetables		0.45a	0.35a	0.03a	0.03a	0.03b	0.02b	0.05a	0.04a	0.07a	0.05a	0.01a	0.01a	0.01a	0.01a	0.65b	0.50b
Tuber vegetables		0.93a	0.72a	0.04a	0.03a	0.02b	0.01b	0.10a	0.08a	0.09a	0.07a	0.01a	0.01a	0.01a	0.01a	1.20ab	0.93ab

Different lower-case letters represent significant differences ($p < 0.05$).

References:

(1) Wang, J. J.; Bowden, R. D.; Lajtha, K.; Washko, S. E.; Wurzbacher, S. J.; Simpson, M. J. Long-term nitrogen addition suppresses microbial degradation, enhances soil carbon storage, and alters the molecular composition of soil organic matter. *Biogeochemistry* **2019,** *142*, 299–313.

(2) Ye, Q. H.; Zhang, Z. T.; Liu, Y. C.; Wang, Y. H.; Zhang, S.; He, C.; Shi, Q.; Zeng, H. X.; Wang, J. J. Spectroscopic and molecular-level characteristics of dissolved organic matter in a highly polluted urban river in South China. *ACS Earth and Space Chemistry* **2019,** *3*, 2033-2044.

(3) Cao, C.; Yang, Y.; Kwan, M. P.; Ma, Z. B.; Karthikeyan, R.; Wang, J. J.; Chen, H. Crop selection reduces potential heavy metal(loid)s health risk in wastewater contaminated agricultural soils. *Science of the Total Environment* **2022,** *819*, 152502.
